# Sequential changes in intraocular pressure during strabismus surgery in patients with thyroid eye disease

**DOI:** 10.1186/s12886-022-02352-8

**Published:** 2022-03-18

**Authors:** Areum Jeong, Won Jae Kim

**Affiliations:** Department of Ophthalmology, Yeungnam University College of Medicine, 170, Hyeonchung-ro, Nam-gu, Daegu, 42415 South Korea

**Keywords:** Thyroid eye disease, Strabismus, Intraocular pressure, Surgery

## Abstract

**Background/Aims:**

To investigate the sequential change in intraocular pressure (IOP) during strabismus surgery in patients with thyroid eye disease (TED).

**Methods:**

This prospective study included patients with TED who underwent strabismus surgery (medial rectus [MR], inferior rectus [IR], and superior rectus [SR] recession) between March 2018 and December 2020. The IOP was measured six times during surgery (5 min after intubation, after isolation of the muscle using a hook and dissection of the surrounding tissue, immediately before muscle detachment, immediately after muscle detachment, after reattachment of the muscle, and after closure of the conjunctiva).

**Results:**

Thirty-five eyes of 18 patients were included. The mean IOP at first was 21.1 mmHg, which significantly increased to 28.6 mmHg after muscle isolation. The IOP significantly decreased to 15.5 mmHg after muscle detachment. This increased to 19.1 mmHg after muscle reattachment. The last IOP was 18.9 mmHg. There were similar patterns of sequential change in the IOP among the three muscles. The MR showed the highest increase in IOP. The IR showed the lowest mean IOP compared with the other two muscles.

**Conclusions:**

The IOP was elevated during the isolation and dissection of the surrounding muscle tissue, especially in the MR. The IOP significantly decreased after muscle detachment and was maintained until the last measurement, even after muscle reattachment. IR showed the lowest IOP among the three muscles during surgery.

## Background

Surgical procedures involving the eyeball and its surrounding tissue could influence the change in intraocular pressure (IOP) during the postoperative period [1, 2]. A previous study showed that IOP significantly declined immediately after cutting the extraocular muscles (EOM) during strabismus surgery in patients with intermittent exotropia [2]. The eyeball of patients with restrictive myopathy might be more vulnerable to IOP change during strabismus surgery. More force and manipulation are required to expose the restrictive muscle during strabismus surgery, which leads to a significant IOP change.

Thyroid eye disease (TED) is the most common type of restrictive myopathy in adult patients [3–5]. TED results in a restrictive myopathy throughout the inflammatory infiltration in the EOM. As a result of progressive fibrosis of the EOM, strabismus and binocular diplopia may be ensured [5, 6]. Previous studies have demonstrated significant IOP reduction in the early postoperative period in patients with TED [7–9]. Understanding the pattern of IOP change during strabismus surgery might be helpful in determining the mechanism of postoperative IOP reduction in patients with TED. In addition, there is a possibility of a variability of IOP during measurement in patients with TED. This is because the eye with TED might require more effort to maintain the primary position during IOP measurement because of the restrictive muscle [6]. This study aimed to investigate the sequential changes in IOP during strabismus surgery in patients with TED.

## Methods

This prospective study included patients with TED who underwent strabismus surgery between March 2018 and December 2020. The present study adhered to the tenets of the Declaration of Helsinki and was approved by the Institutional Review Board of Yeungnam University Hospital. All participants provided written informed consent. Individuals with a previous history of strabismus, ocular surgery including orbital decompression, those previously diagnosed with glaucoma, and those under medication were excluded from this study.

### Patient’s preoperative evaluation and strabismus surgery

All patients underwent full ophthalmologic examination, including testing for visual acuity and ocular alignment status, slit-lamp biomicroscopy, and fundus examination. The angle of deviation was measured using an alternate prism cover test at 6 m and 33 cm fixation. The patients’ strabismus conditions were considered stable for at least 3 months before surgery. All patients underwent computed tomography imaging one day before the surgery. All surgeries were performed under general anesthesia by a single surgeon (WJK). Strabismus surgery was performed using the surgical dosage of our clinic based on the patients’ angle of deviation measured the day before the surgery. The surgical procedure was conducted using a limbal incision. The operated muscle underwent a conventional recession procedure without adjustable sutures. Surgical procedures for strabismus performed on all patients consisted of bilateral medial rectus (MR) recession, bilateral inferior rectus (IR) recession, unilateral IR recession, and contralateral superior rectus (SR) recession.

### Measurements of IOP during strabismus surgery

The IOP was measured six times during surgery using handheld tonometry (Tono-Pen XL, Medtronic Solan, USA) by the same surgeon. The median of three consecutive readings was taken. The phases of six sequential measurements were as follows.

Phase 1: first measurement (5 min after tracheal intubation), Phase 2: after isolation of the operated muscle using a hook and dissection of the surrounding tissue, Phase 3: immediately before the detachment of the operated muscle (after securing the muscle with 6–0 Vicryl sutures), Phase 4: immediately after the detachment of the operated muscle, Phase 5: after the reattachment of muscle directly to the sclera; Phase 6: last measurement (after the closure of the conjunctiva). All measurements were taken in the same body position.

### Statistical analysis

Continuous data are expressed as mean with standard deviation, and categorical data are expressed as counts and percentages. The paired t-test was used to compare the IOP change between the different measurements. The repeated measures ANOVA (rmANOVA) test with post hoc Bonferroni test was used to analyze the differences in IOP according to the muscle operated on. Data were analyzed using SPSS version 20.0 (IBM Corporation, Armonk, NY, USA). A *p*—value of < 0.05 was regarded as statistically significant.

## Results

### Basic characteristics and ocular findings of included patients with TED

During the study period, 19 patients with TED were recruited. Among these, three eyes of two patients were excluded from the study due to difficulties encountered during surgery. Thirty-five eyes of 18 patients (6 female) were included in this study. The basic characteristics of the included patients are shown in Table 1. The mean interval from initial visit to surgery was 15.3 months (range: 3–67 months). Approximately half the patients (55.6%, 10/18) had hyperthyroidism. Strabismus and binocular diplopia initially presented with thyroid dysfunction in six patients (33.3%, 6/18). Nine patients (50.0%, 9/18) were active or ex-smokers, and all of them were male. The ocular findings are listed in Table 2. Most included patients had lid abnormalities or proptosis. Hypotropia was the most common type of strabismus. Two patients showed esotropia and hypotropia. Among these, one underwent bilateral MR recession and the other underwent bilateral IR recession. The mean preoperative ocular deviation was 35.00 prism diopters (PD) in esotropia and 31.14 PD in hypotropia. The mean IOP measured one day before surgery was 17.2 mmHg in the right eye and 18.1 mmHg in the left eye (*p* = 0.636, Mann–Whitney U test).

**Table 1:** Basic characteristics of included patients with thyroid eye disease

35 eyes with 18 patients with thyroid eye disease	
Mean age at initial visit (range), yr	56.8 ± 10.1 (35–74)
Gender (Male: Female)	12: 6
Mean age at surgery (range), yr	58.3 ± 10.2 (36–74)
Interval from initial visit to surgery (range), mo	15.3 ± 18.1 (3–67)
Underlying systemic disease, n (%)	
DM	2 (11.1)
Hypertension	1 (5.6)
Both DM and hypertension	1 (5.6)
Thyroid dysfunction, n (%)	
Hyperthyroid	10 (55.6)
Euthyroid	6 (33.3)
Hypothyroid	2 (11.1)
Smoking, n (%)	
Active smoker	5 (27.8)
Ex-smoker	4 (22.2)
Non-smoker	9 (50.0)
Spherical equivalent refractive errors, D	
Right eye, 18 eyes	-0.97 ± 2.30 (-6.38 to 1.50)
Left eye, 17 eyes	-0.71 ± 2.20 (-6.25 to 1.50)

**Table 2:** Ocular findings included patients with thyroid eye disease

35 eyes with 18 patients with thyroid eye disease	
Lid retraction or swelling, n (%)	31 (88.6)
Proptosis	31 (88.6)
Ocular motility findings, n (%)	
Esotropia	2 (11.1)
Hypotropia	14 (77.8)
Esotropia with hypotropia	2 (11.1)
Preoperative ocular deviation (range), PD	
Esotropia	35.00 ± 8.94 (25 to 45)
Hypotropia	31.14 ± 8.02 (14–40)
Extraocular muscles enlargement on imaging, n (%)	
No definitive enlargement of operated muscle	11 (31.4)
Enlargement of operated muscle	7 (20.0)
Enlargement of multiple muscles	17 (48.6)
Operated eye (Right: Left)	18: 17
Surgical procedures, n (%)	
MR recession	6 (17.1)
IR recession	16 (45.7)
SR recession	13 (37.1)
Intraocular pressure (mmHg)	
Right eye, 18 eyes	17.2 ± 3.1 (9.0—23.0)
Left eye, 17 eyes	18.1 ± 3.4 (12.0—25.0)

### Sequential changes of IOP during strabismus surgery in patients with TED

**Fig. 1:**
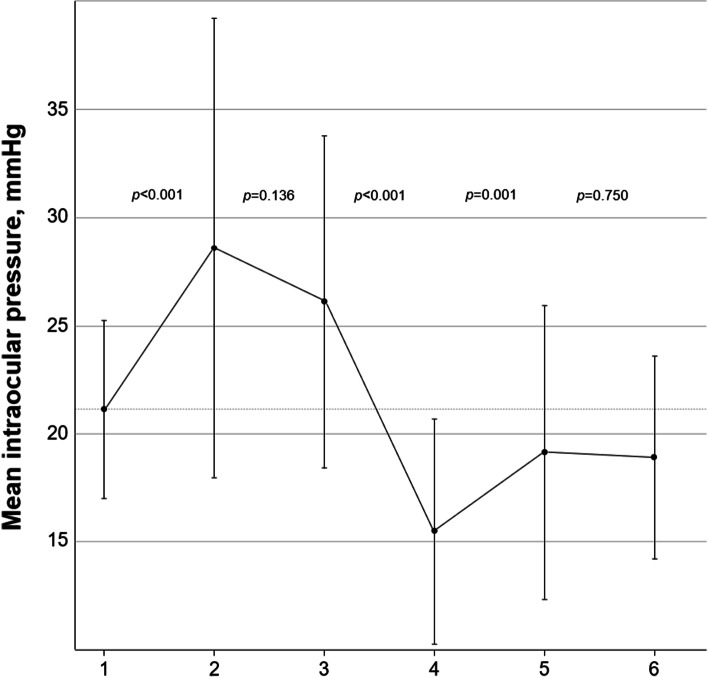
Sequential changes in intraocular pressure (IOP) during strabismus surgery in patients with thyroid eye disease (TED). The mean IOP at the first measurement was 21.1 mmHg, and it significantly increased to 28.6 mmHg after isolation of the operated muscle (*p* < 0.001). Then, there was no significant change in IOP before the detachment of the operated muscle (*p* = 0.136). The mean IOP significantly decreased to 15.5 mmHg after the detachment of the operated muscle (*p* < 0.001). This increased to 19.1 mmHg after the reattachment of the muscle (*p* = 0.001). The mean IOP at the last measurement was 18.9 mmHg (*p* = 0.750). The lower mean IOP compared with first measurement was maintained even after muscle reattachment

The sequential changes in IOP during surgery are listed in Fig. 1. The mean IOP at the first measurement was 21.1 ± 4.1 (range, 15–30 mmHg) and significantly increased to 28.6 ± 10.6 (range, 8–59) mmHg after the isolation of the operated muscle (*p* < 0.001). Then, there was no significant change in IOP before the detachment of the operated muscle (*p* = 0.136). The mean IOP significantly decreased to 15.5 ± 5.2 (range, 7–28) mmHg after the detachment of the operated muscle (*p* < 0.001). These values increased to 19.1 ± 6.8 (range, 8–31) mmHg after muscle reattachment (*p* = 0.001). The mean IOP at the last measurement was 18.9 ± 4.7 (10–33) mmHg (*p* = 0.750). The IOP significantly decreased after the detachment of the muscle. The lower mean IOP compared with first measurement was maintained even after muscle reattachment.

### Differences in IOP changes according to the operated muscle in patients with TED

**Fig. 2:**
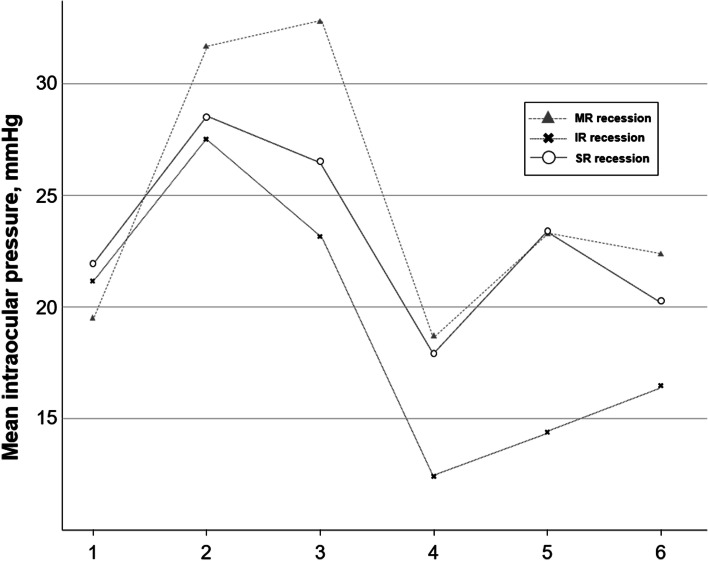
Sequential changes in intraocular pressure (IOP) according to the operated muscle in patients with thyroid eye disease (TED). The surgical procedures for strabismus performed on all patients consisted of medial rectus (MR) recession, inferior rectus (IR) recession, and superior rectus (SR) recession. There were similar patterns of sequential change in the IOP during strabismus surgery among the three muscles. A repeated-measure ANOVA analysis showed statistically significant group-by-time effects for the change of IOP among the three muscles during surgery (*p* < 0.001). The mean IOP of MR were higher during surgery compared with those of other two muscles (*p* = 0.027 with IR recession; *p* = 1.000 with SR recession). The mean IOP of IR were lower during surgery compared with those of other two muscles (*p* = 0.051 with SR recession)

We also analyzed the change in IOP according to the muscle operated on. There were similar patterns of sequential change in the IOP during strabismus surgery among the three muscles (Fig. 2). An rmANOVA analysis showed statistically significant group-by-time effects for the change of IOP among the three muscles during surgery (*p* < 0.001). The mean IOP of MR were higher during surgery compared with those of other two muscles (*p* = 0.027 with IR recession; *p* = 1.000 with SR recession). The mean IOP of IR were lower during surgery compared with those of other two muscles (*p* = 0.051 with SR recession).

## Discussions

Our study is the first to demonstrate sequential changes in IOP during strabismus surgery in patients with TED. The IOP was elevated during the isolation and dissection of the surrounding tissue of the operated muscle. The IOP significantly decreased after the detachment of the operated muscle and was maintained until the last measurement, even after the reattachment of the operated muscle. The IR showed the lowest IOP among the three muscles during surgery.

During strabismus surgery, the eyeball can experience a change in IOP. The manipulation of the eyeball and the surrounding tissue is unavoidable during surgery, and these changes might lead to changes in the IOP. The study by Yoo et al. demonstrated that the IOP significantly declined immediately after cutting an EOM during strabismus surgery, and these changes were positively correlated with the constancy of exodeviation [2]. However, their study only included patients with intermittent exotropia and suggested further study including patients with restrictive strabismus.

There is an association between elevated IOP and TED [7–11]. Previous studies demonstrated that strabismus surgery resulted in a significant reduction in the IOP in the early postoperative period in patients with TED [7, 11]. However, no study has evaluated the sequential change in IOP during strabismus surgery in patients with TED. We were interested in where and when the IOP significantly decreased during strabismus surgery. In addition, the possibility of IOP elevation is also considered because the eyeball in restrictive strabismus might be more vulnerable to IOP change, in both increasing and decreasing IOP during strabismus surgery [6]. Experiencing elevated IOP by finger palpation during strabismus surgery might not be uncommon in patients with TED, the most common type of restrictive strabismus in adults.

In this study, the IOP significantly increased during the isolation of the muscle and dissection of the surrounding tissue of the operated muscle. Among the three muscles, MR showed the largest increase in mean IOP. This might be because the severe restriction in the operated muscle in patients with TED required more manipulation and force to expose the muscle. These also led to an increase in the IOP during strabismus surgery. The pulled-in-two syndrome, the sudden rupture of an extraocular muscle during strabismus surgery, usually involves MR and IR [12]. TED is one of the most common underlying risk factors [12]. These findings are consistent with our results. We postulated that the MR had limited orbital space to access compared with other recti muscles, hence requiring more force to expose the muscle and more vulnerable to a significant elevation of the IOP during the procedure [13]. Surgeons should be aware of the possibility of IOP elevation during strabismus surgery in patients with TED, especially during MR recession.

The IOP significantly decreased after the detachment of the muscle, and these changes were maintained until the last measurement during surgery. The lower mean IOP compared with first measurement was maintained even after muscle reattachment. All three muscles showed similar patterns of IOP changes. The study by Gomi et al. demonstrated significant IOP reduction in the early postoperative period in patients with TED [11]. Their study showed that the largest decrease was observed in the eye that had the IR muscle recessed. These findings are similar to our results, in which IR showed the lowest IOP among the three muscles during surgery. Increased episcleral venous pressure resulting from orbital congestion and venous outflow obstruction, increased resistance to trabecular outflow, and restriction and compression of the globe by fibrotic and enlarged rectus muscle were the supposed mechanisms of IOP elevation in patients with TED [8, 11]. A previous study demonstrated that orbital decompression and strabismus surgery resulted in a significant reduction in the IOP during the early postoperative period, but not in cases treated with orbital radiation [8]. The reduction in IOP after orbital decompression might be caused by a surgical expansion of the orbital volume. However, the mechanism of IOP reduction after strabismus surgery remains unclear. Several patients with tight IR caused by TED have their eyes fixed in downgaze, and they have to exert extra force to attain the primary position because of the tethering effect of fibrotic and shortened extraocular muscles [10]. There may be variability in IOP during measurement caused by eyeball position [7]. However, these possibilities might be minimized in this study because we measured the IOP using Tono-Pen under general anesthesia. There was no extra effort required to maintain the primary position during IOP measurement in our study.

We postulated that both reposition and relief of restriction of fibrotic muscle might lead to a decrease in IOP, which is maintained during strabismus surgery. Contractures of the EOM lead to an increase in the IOP. The EOM works in a coordinated fashion, maintaining significant tension or “tonus,” even when the eye is in the primary position and, thus, presumably “at rest.” Therefore, the sum of these effects of 6 EOMs might influence the change in IOP. The study by Hayashi et al., which included patients with highly myopic strabismus, demonstrated a reduction in IOP after muscle union surgery [14]. They suggested that abnormal orbital anatomy such as displacement of the recti muscles and a large globe size compared to the orbital volume may be responsible for the changes in the IOP. In cases of TED, the displacement of the EOM and an enlarged EOM size compared to the orbital volume result in changes in IOP. In addition, Ghosh et al. demonstrated that the angle of gaze has a significant effect on axial length, with the greatest elongation occurring in inferonasal gaze [15]. The elongation of the eye appears to be due to the influence of the EOM. Although their study did not measure the IOP, the elongation of the eye might influence IOP change, especially in the inferonasal gaze, the main function side of the IR and MR. The MR or IR recession, a weakening procedure of the EOM, reduced these effects and reduced the IOP. Therefore, in patients with TED, reposting of the extraocular muscles caused by strabismus surgery might improve orbital anatomy, leading to a reduction in IOP.

This study had some limitations. First, the change in IOP according to surgical amount was not evaluated because of the small number of included patients. A previous study showed that there is a direct linear correlation between the amount of hypotropia as measured in PD and the increase in IOP during upgaze [10]. There would be an association between IOP change and surgical amount. Second, the depth of general anesthesia and the device used in the study can affect the result of IOP measurement. The various physiologic parameters that may be altered during general anesthesia can influence the IOP [16]. The study by Rahman et al. revealed that there was no significant difference of measurement between Topo-Pen XL and Goldmann applanation tonometer in patient with TED [17]. Further studies are warranted to evaluate the changes of IOP with various measurement devices under more controlled anesthetic depth. Third, cutting the ciliary artery during detachment may influence the change in IOP [14]. When the recti muscles are detached from the sclera, cutting the anterior ciliary arteries reduces the production of aqueous humor, resulting in a decrease in the IOP. However, these effects are minimal. Because only one rectus muscle of each eye was operated on in the included patients.

## Conclusions

In conclusion, there was a significantly sequential changes in IOP during strabismus surgery in patients with TED. The IOP was elevated during the isolation and dissection of the surrounding tissue of the operated muscle. The IOP significantly decreased after the detachment of the operated muscle and was maintained until the last measurement, even after the reattachment of the operated muscle. The IR showed the lowest IOP among the three muscles during surgery.

## Data Availability

All data generated or analysed during this study are included in the manuscript.

## Abbreviations

IOP: Intraocular pressure
EOM: Extraocular muscles
TED: Thyroid eye disease
MR: Medial rectus muscle
IR: Inferior rectus muscle
SR: Superior rectus muscle
